# Research Progress on New Functions of Animal and Plant Proteins

**DOI:** 10.3390/foods13081223

**Published:** 2024-04-17

**Authors:** Hao Duan, Gaigai Liu, Duo Feng, Zhuoye Wang, Wenjie Yan

**Affiliations:** 1 College of Biochemical Engineering, Beijing Union University, Beijing 100023, China; dhuanao@163.com (H.D.); lgg010403@163.com (G.L.); 2 Beijing Key Laboratory of Bioactive Substances and Functional Food, Beijing Union University, Beijing 100023, China; 2022050352049@buu.edu.cn; 3 Institute of Food and Nutrition Development, Ministry of Agriculture and Rural Affairs, Beijing 100081, China; 15525926785@163.com

**Keywords:** plant proteins, animal proteins, functional foods, current status of applications, new functions, development and research

## Abstract

Protein is composed of peptides, essential nutrients for human survival and health, and the easy absorption of peptides further promotes human health. According to the source of the protein, it can be divided into plants, animals, and micro-organisms, which have important physiological effects on the health of the body, especially in enhancing immunity. The most widely used raw materials are animal protein and plant protein, and the protein composition formed by the two in a certain proportion is called “double protein”. In recent years, China’s State Administration for Market Regulation has issued an announcement on the “Implementation Rules for the Technical Evaluation of New Functions and Products of Health Foods (Trial)”, which provides application conditions and listing protection for the research and development of new functions of health foods. At present, some researchers and enterprises have begun to pay attention to the potential of animal and plant proteins to be used in new functions. In this article, the research progress of animal and plant proteins in the new functions of Chinese health food is reviewed in detail, and suggestions for future research on animal and plant proteins are put forward.

## 1. Introduction

Animal and plant proteins provide essential support for human health, with the former mainly derived from meat, eggs, or milk of edible animals such as poultry, livestock, and fish, and the latter mainly from legumes, cereals, or nuts. Generally, animal proteins are more nutritious and easily absorbed by the human body compared to plant proteins. Still, animal protein intake leads to cholesterol and saturated fatty acid intake at the same time [1], and the long-term overconsumption of animal proteins can easily lead to health problems. A study involving 70,696 participants found that high animal protein intake was positively associated with cardiovascular mortality, while high plant protein intake was negatively associated with all-cause and cardiovascular mortality, and that the replacement of animal protein by plant protein, especially processed red meat, was associated with lower mortality rates, which suggests the importance of the protein source [2]. Therefore, some studies have proposed a strategy of replacing animal proteins with plant proteins to improve human health, while, at the same time, reducing the number of animals raised and slaughtered, and helping to reduce the environmental pollution caused by animal husbandry and the degradation of grasslands caused by overgrazing. However, it is difficult to advance this strategy because plant proteins and animal proteins contain different levels of amino acids and other nutrients, as well as contaminants, and, therefore, have different strengths and weaknesses. In contrast to plant proteins, animal proteins provide the optimal levels of all of the nine essential amino acids our body needs, making them “complete proteins”, whereas plant proteins do not contain them all within one source. Animal proteins are often associated with a better taste and palatability than vegetable proteins and also provide a higher nutrient density than plant proteins; a lot of iron, which is much more absorbent than the iron found in vegetables; and Vitamin B_12_, which is not naturally sourced from plants [3]. Moreover, protein hydrolysis can release biologically active peptides to promote health. In recent years, China’s State Administration for Market Regulation issued the announcement of “Implementation Rules for Technical Evaluation of New Functions and Products of Health Food (for Trial Implementation)”, which provides reporting conditions and listing protection for the research and development of new functions of health food, and this strategy greatly promotes the research and development enthusiasm of raw materials with new functions of health food and effectively promotes the development of whole health food and functional food ingredients. Therefore, some researchers and enterprises have already started to pay attention to the potential of protein raw materials applied in new functions. This article provides a detailed overview of the research progress of animal and plant proteins in new functions and puts forward suggestions for animal and plant proteins in future research.

## 2. Progress in the Study of New Functions of Animal and Plant Proteins

### 2.1. Helps Fight Skin Photoaging

**Figure 1:**
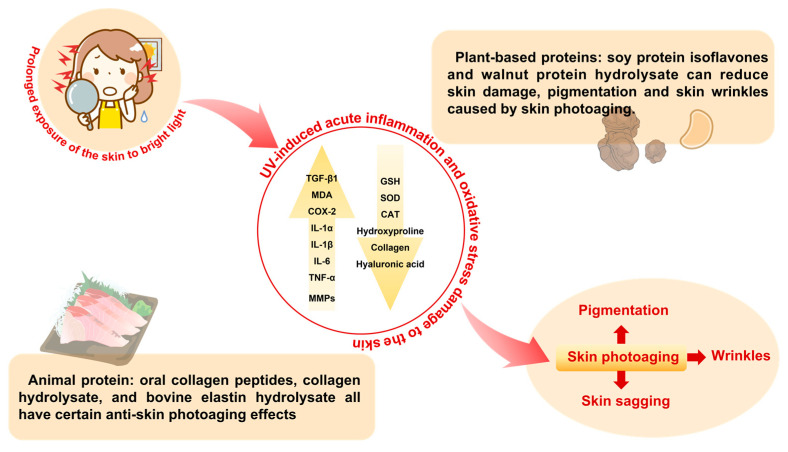
Causes of skin photoaging.

Skin photoaging, caused by continuous exposure to ultraviolet radiation, is one of the main causes of skin aging. The main manifestations of skin photoaging are wrinkles, sagging, and pigmentation [4]. Figure 1 illustrates the main causes of skin photoaging. Studies have confirmed that ultraviolet light can cause acute inflammation and oxidative stress damage to the skin, resulting in the significant upregulation of related biomolecules such as transforming growth factor β1 (TGF-β1), malondialdehyde (MDA), pain mediator cyclooxygenase-2 (COX-2), interleukin immune-regulators including IL-1α, IL-1β, IL-6, and inflammatory cytokine, namely, Tumour Necrosis Factor alpha (TNF-α), and the significant downregulation of antioxidants like glutathione (GSH), superoxide dismutase (SOD), catalase (CAT), etc. [5,6,7,8,9]. It also induces a significant increase in MMPs in skin fibroblasts [10]. In particular, Matrix Metalloproteinase-1 (MMP-1), which easily binds to type I collagen (Col I) and type III collagen (Col III), leads to a large amount of degradation of collagen fibres [11] and promotes the hyperplasia and hypertrophy of the stratum corneum [12], which is also a key indicator of anti-skin photoaging research. In addition, the upregulation of other members of MMPs, such as MMP-3 and MMP-9, is a damaging substance that induces decreased skin elasticity and collagen synthesis, resulting in photoaging of the skin [13,14].

Studies have shown that soy protein isoflavones in plant proteins can help improve skin photoaging, including reducing wrinkles and pigmentation and improving skin hydration [15]. In addition, it was also found that walnut protein hydrolysate had a significant protective effect on UV-induced skin photoaging damage in Sprague–Dawley (SD) rats by oral administration for 18 weeks, and the mechanism was mainly by regulating the decomposition of extracellular matrix (ECM) components, increasing Col I, Col III, hydroxyproline (Hyp), and hyaluronic acid (HA), as well as the downregulation of MMP-1 activity and restoration of the elastic structure of the ECM. Among them, Hyp is a relatively stable substance in collagen (13.4%), so its content determination can be used as a reliable method to measure the ability of cells and tissues to synthesise collagen [16]. In studies on the anti-photoaging of animal proteins, it has been found that the anti-skin photoaging effect of collagen has been widely recognised [17,18,19,20]. In particular, fish collagen has been the most studied [21]. At the same time, animal protease hydrolysate also has a significant anti-skin-photoaging effect. The pentapeptide WNLNP purified from oyster protease hydrolysate has a significant anti-photoaging effect on the UVB-irradiated HaCaT (human immortalised keratinocytes) photoaging cell model. There is a dependent relationship between the anti-photoaging effect and the dose, suggesting that its mechanism of action may be by inhibiting ROS production, decreasing MMP-1 expression, and increasing extracellular procollagen I content to exert a protective effect [22]. In addition, animal proteins such as oral collagen peptides [20], collagen hydrolysate [19], and bovine elastin hydrolysate [23] all have certain anti-skin-photoaging effects. At present, many studies support the anti-skin-photoaging of animal proteins and their peptides, especially collagen and its peptides, and the mechanism is also clear. This suggests that animal proteins and their peptides may be more advantageous for improving skin health.

### 2.2. Helps Reduce High Uric Acid

**Figure 2:**
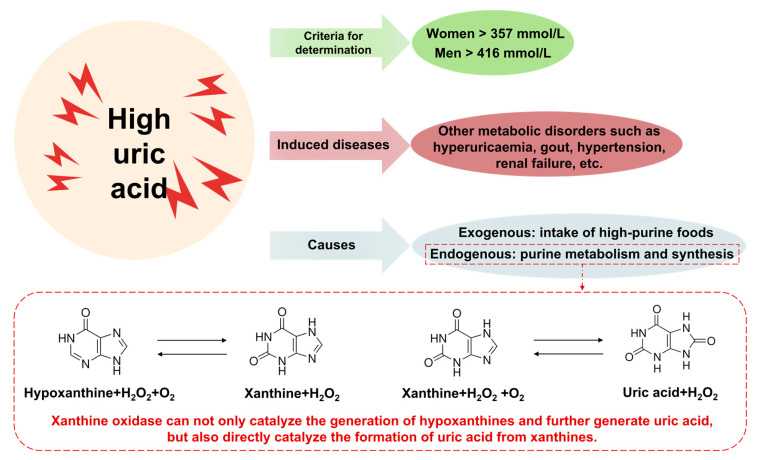
Criteria for evaluation of hyperuricaemia, induced associated diseases, and endogenous uric acid metabolic pathways.

The end product of purine oxidation is uric acid (UA), which is more likely to induce hyperuricemia (HUA) when the uric acid is too high (357 mmol/L for women >> and 416 mmol/L for men), and the probability of hypertension is increased by 13 percent for every 1 mg/dL increase in serum uric acid [24]. In more severe cases, renal impairment may occur [25], and other metabolic disorders. In addition, HUA is closely associated with gout, with more than 90 percent of gout patients presenting with high UA [26]. Studies have shown that there are two main sources of uric acid in the body: one is the dietary pathway for the nucleotide decomposition of nucleoprotein-rich foods, and the other is the endogenous pathway formed by the synthesis of small molecule compounds such as amino acids and ribose phosphate and nucleic acid decomposition in the body. The endogenous pathway is the main pathway [27]. Therefore, reducing endogenous uric acid production is the main strategy to reduce high uric acid [28]. Among them, adenosine deaminase (ADA) and xanthine oxidase (XOD) are key enzymes in the endogenous synthesis pathway of UA [29]. ADA catalyses the decomposition of adenosine to inosine, which is further converted to hypoxanthine, and, finally, catalysed by XOD to obtain UA [30]. XOD catalyses the generation of UA from xanthines or catalyses the generation of hypoxanthines to xanthines, and then further to UA [31]. Therefore, ADA inhibitors and XOD inhibitors are currently the main means used to reduce UA. Figure 2 shows the evaluation criteria for high uric acid, the related diseases induced, and the causes of it.

Clinical studies have shown a significant correlation between protein intake levels and serum urea, uric acid, and serum creatinine levels [32]. While some animal and plant proteins show better uric-acid-lowering effects. Previous studies have found that dietary peptides mainly play a role in reducing UA by inhibiting XOD activity, regulating the expression level of UA transporters, and regulating intestinal microbiota [33]. Animal and plant protein hydrolysates also play a significant role in reducing UA, such as walnut protein hydrolysate [34], tuna protein hydrolysate [35], and whey protein hydrolysate [36]. In addition, the combination of animal and plant proteins may have a superior UA-lowering effect. It has been found that the combination of rice peptide and collagen peptide can significantly inhibit the activity of XOD and ADA, effectively reduce UA, and exert a certain nephroprotective effect, and the combined use of the combined UA-lowering effect is significantly better than that of the single administration group, suggesting that the combination administration can synergistically enhance excessive UA excretion [37]. These studies have shown that proteolysis has a significant role in reducing uric acid levels and that the combination of animal and plant proteins is significantly more effective than a single proteome.

### 2.3. Anti-Colorectal Cancer

**Figure 3:**
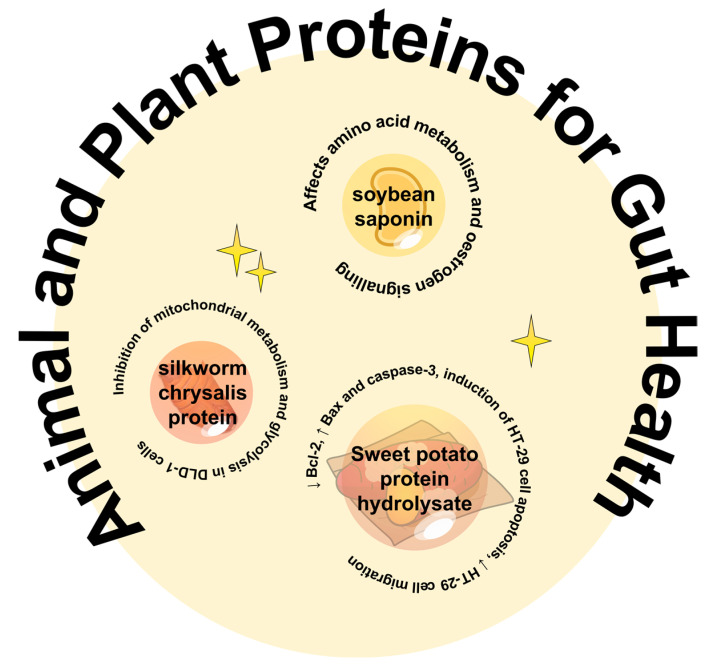
The potential of protein-based raw materials in colon cancer.

Colorectal cancer is a common malignancy of the colon of unknown cause, which is related to dietary factors, colon polyps, and chronic colitis, and is common in the male population [38]. The number of colorectal cancer patients is increasing year by year due to the characteristics of cancer, characterised by cell replication immortality, increased proliferation, invasion and metastasis, the evasion of tumour suppressor genes, angiogenesis, and resistance to apoptosis [39]. Figure 3 summarises the application potential of protein-based raw materials in colon cancer.

It has been reported that bean saponin I extracted from legumes does not affect the colon cancer cell lines HCT116 (IC_50_ = 161.4 μM) and LoVo (IC_50_ = 180.5 μM) [40]. Network pharmacology reveals nine potential targets in the treatment of colon cancer were discovered, and a total of 45 differential metabolites were identified through metabolomic analysis, among which the KEGG pathway was mainly enriched in amino acid absorption and metabolism-related pathways, indicating that the anti-colon cancer effect of thet bean saponin I extracted may be played by affecting amino acid metabolism and the estrogen signalling pathway [40]. Proteolytic raw materials play an important role in the prevention and treatment of colon cancer [41]. It has been found that six kinds of sweet potato protein hydrolysate prepared by using six kinds of protease have a certain anti-proliferative effect on HT-29 colon cancer cells, and the sweet potato protein hydrolysate prepared by Alcalase has the highest anti-proliferative effect, and the component with a molecular weight of less than 3 kDa can significantly cause G2/M cell cycle arrest, increase the expression of p21, and reduce the expression of Bcl-2, increasing Bax expression and inducing caspase-3 activation-induced apoptosis in HT-29 cells and inhibiting the migration of HT-29 cells, suggesting the potential of sweet potato protein hydrolysate prepared from Alcalase with a molecular weight of less than 3 kDa in the fight against colon cancer [42]. In addition to the anti-colon cancer effects observed in plant proteins, a significant value of insect proteins in anti-colon cancer was also found. Studies have shown that silkworm pupa protein has a certain positive effect on the proliferation, apoptosis, and energy metabolism of human colon cancer DLD-1 cells, which is manifested in the fact that silkworm pupa protein can significantly inhibit the proliferation of DLD-1 cells, promote apoptosis, inhibit mitochondrial metabolism and the glycolysis of DLD-1 cells, and reduce cellular energy metabolism, indicating that silkworm pupa protein has the potential to be used as an anticancer drug [43]. Further validation results from animal experiments showed that the subcutaneous tumorigenesis method was used to verify the therapeutic effect of SPP on colon cancer nude mice in vivo. The results showed that silkworm pupa protein exerted significant anti-colon cancer activity by decreasing the levels of TNF-α and IL-2, increasing the level of INF-γ, reducing oxidative stress in the liver, and regulating the level of serum inflammation [44]. The above studies show that the application potential of animal and plant proteins in the fight against colorectal cancer is greater, and more raw material mining is worth discussing in the future.

### 2.4. Regulates Intestinal Health

The intestine is an important digestive organ of the body, and the nutrients in food are mainly absorbed in the intestine. At the same time, the intestinal tract is also an important immune organ; its natural barrier maintains the integrity of the intestinal epithelium, can prevent the invasion of pathogenic micro-organisms, and can also regulate the secretion of antibodies by the intestinal mucosa to act on the intestinal immune system, and further affect innate immunity and acquired immunity. In addition, the gut also contains a large number of nerve cells and microbiota, which is why the gut is also known as the “second brain” [45]. The dysbiosis of the intestinal microbiota will not only damage the intestinal health, but also cause the development of animals to be hindered; the function of the digestive tract will decline, and will lead to a variety of sub-health conditions [46], so intestinal health is an important guarantee for the health of the body. Proteins from different food sources contain different amounts of nutrients that interact with the microbiota in the gut, which, in turn, affects the host metabolism and gut health.

The effect of protein intake on the gut microbiota has been demonstrated [47], especially for plant proteins, which have a positive effect on gut microbioregulation due to their important bioactive peptides. Soy protein has been shown to improve the growth of early malnourished rats by modulating the gut microbiota and metabolites in the colon and serum [48]. In addition, soy protein intake led to increased microbiota diversity, particularly the production of short-chain fatty acids, which lowered cecal pH. It was also found that morning soy protein intake had a greater effect on the microbiota [49]. At the same time, the fermented plant protein raw materials will further promote its role in regulating the intestinal flora. A six-week treatment of fermented soybean protein on spontaneously hypertensive rats was found to significantly reduce systolic blood pressure and diastolic blood pressure, inhibit serum ACE activity, increase serum superoxide dismutase activity and nitric oxide level, and reduce serum malondialdehyde levels, and a 16S rRNA analysis showed that fermented soybean protein could significantly increase the microbial richness and diversity of model animals, and significantly reduced the Firmicutes/Bacteroidetes ratio, increased propionate- and H_2_S-producing bacteria, and reduced *Streptococcaceae* and *Erysipelotrichales* levels, suggesting that fermented soy protein has the potential to positively modulate the intestinal microbiota and alleviate hypertension [47]. In addition to soy protein, lactoferrin has been shown to modulate metabolic disorders by modulating the gut microbiota pathway in high-fat diet mice [50]. Altogether, the studies have shown that plant protein has a significant effect on the regulation of intestinal flora, and can improve other diseases of the body through the regulation of intestinal flora.

### 2.5. Contributes to Ovarian Health

Ovarian cancer can be subdivided into more than five distinct histological subtypes with different identifiable risk factors, cells of origin, molecular composition, clinical features, and treatment strategies. Among them, genetic factors, age, and the use of postmenopausal hormone therapy are unfavorable factors for the increase of ovarian cancer, and ovarian cancer is often diagnosed at an advanced stage, and there is a lack of effective screening strategies [51]. Among them, epithelial ovarian cancer is the most common type of ovarian cancer [52]. Treatment options for ovarian cancer generally include chemotherapy, targeted therapy, immunotherapy, radiation therapy, and hormone therapy. Nevertheless, these regimens demonstrate greater side effects and harm to the body, and most patients relapse with chemotherapy resistance after receiving conventional therapy [53]. Therefore, certain nutritional intervention strategies can be used as an auxiliary in the treatment of ovarian cancer patients, which will help to speed up the recovery of the body after surgery, enhance the efficacy of radiotherapy and chemotherapy, reduce adverse reactions, prolong survival, and improve the quality of life.

Insulin-like growth factors (IGFs) have been implicated in the development of a variety of cancers, and the IGF signalling system consists of insulin, IGF1, IGF2, and insulin-like growth factor-binding proteins (IGFBPs) [54]. Among them, the elevated expression and activity of IGF1R are associated with the occurrence of ovarian cancer, and the growth of ovarian tumours can be effectively inhibited by the use of blocker antibodies or tyrosine kinase inhibitors [55]. Studies have shown that high levels of plant protein intake can reduce the growth of human HM-1 and ID-8 ovarian cancer cells in mice, possibly through the relative inhibition of the IGF/Akt/mTOR pathway [56]. In addition, the HSP90AB1 gene associated with ovarian cancer is a member of the 90 family of heat shock proteins involved in signal transduction, protein folding and degradation, and morphological evolution [57]. HSP90AB1 overexpression has been found to play an important role in cisplatin resistance in ovarian cancer, and maggot extracts (the larvae of *Lucilia sericata*) can enhance the sensitivity of ovarian cancer cells to cisplatin toxicity by reducing HSP90AB1 expression and increasing cisplatin-induced DNA damage [58]. It can be seen that protein raw materials may play a certain role in the adjuvant treatment of ovarian cancer.

### 2.6. Other New Functional Research Directions

With the development of society, people are facing increasing mental stress, and the number of people who suffer from anxiety and depression is also increasing [59], so there is a high demand for products that improve emotional health. Bioactive peptides extracted from egg white [60], salmon [61], bovine casein [62], soybean [63], and tilapia skin [64] have been shown to have antidepressant/anxiety activity. In addition, the compatibility of protein raw materials with other raw materials showed more obvious antidepressant/anxiety activity, and, after the combination of casein hydrolysate and γ-aminobutyric acid (GABA) in the anxiety/depression model mice (4:1 compatibility ratio), it was observed that the complex could increase the levels of GABA and 5-HT in the brain of mice, and the histopathological status of the CA3 region of the hippocampus of mice was also significantly improved [65].

Oral ulcers are common in daily life and tend to occur on the lips, tongue, and floor of the mouth, and are mainly manifested as oral mucosal pain and bleeding, which affect people’s eating, cause serious distress to life, and have recurrence [66]. Ulcer healing has been shown to include phases of inflammation, fibrosis, and tissue remodeling [67,68]. As a major component of the extracellular matrix, collagen has been reported to be directly involved in and regulate fibrosis and tissue remodeling [68]. Marine collagen peptides have been found to have a good application in skin wound healing [69,70,71], and the administration of tilapia skin marine collagen peptide to oral ulcer model mice can significantly reduce the infiltration of inflammatory cells at the oral ulcer site of the model mice, downregulate the expression of TNF-α and IL-1β, and effectively promote fibrogenesis, angiogenesis, and collagen formation, indicating that the improvement effect of tilapia skin marine collagen peptide on oral ulcers mainly begins to play a role in the middle and late stages of healing [72]. At present, there are few studies on the application of protein raw materials in oral ulcers, and the existing research also focuses on marine collagen peptides, which suggests that the mining of protein raw materials and the study of the mechanism of action should be more in-depth in future research. In addition, an increased intake of plant protein rather than animal protein has been found to have a better preventive effect on the development of chronic kidney disease [73].

Combinedly, protein raw materials, especially animal and plant protein hydrolysates or peptides, demonstrated that they have good application value in new functions such as skin health, high uric acid, the adjuvant treatment of cancer, oral health, and emotional health. This suggests that the research on the deep processing of protein raw materials will help to give more possibilities for the original application direction of proteins.

## 3. Opinions and Suggestions on the Research and Development of Animal and Plant Proteins in New Functional Products

**Figure 4:**
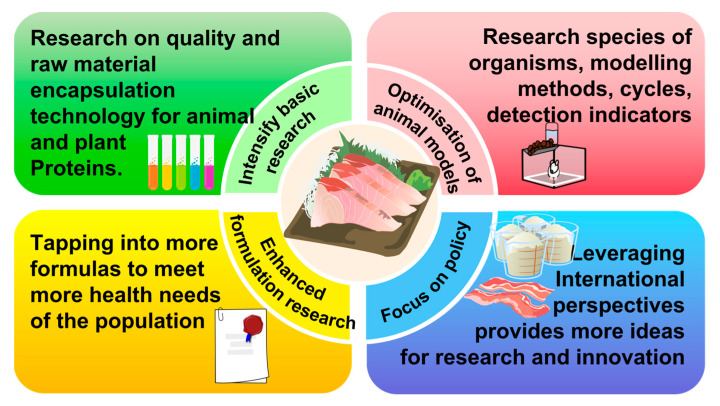
Summary of recommendations for future research on animal and plant proteins.

Proposals for future research on animal and plant proteins are detailed in Figure 4.

### 3.1. Strengthening Basic Research on Animal and Plant Proteins and Peptides

Proteins are important for animal growth and development and can provide many health benefits to humans, especially through the intake of plant and animal proteins. However, protein raw materials have different nutrients and contaminants depending on their source, and these unfavourable factors should be fully considered and dealt with before their application, which requires a large amount of basic research information to support these works.

The first thing to pay attention to is the quality of animal and plant protein, as it is an important indicator of nutritional adequacy and health of the body, which refers to the bioavailability, digestibility, and amino acid composition of the animal and plant protein [74]. The study of bioavailability involves the release, absorption, distribution, metabolism, and excretion of food matrix after ingestion, and its main evaluation methods are divided into an in vitro digestion model and an in vivo animal model. The in vitro digestion model has the advantages of a simple process, low cost, 3R principle, short cycle, and no ethical restrictions, among which the gastrointestinal digestion model and the oral-gastrointestinal–intestinal digestion model are the two main models [75,76]. To a certain extent, in vitro digestion experiments can reflect the complex physicochemical and physiological changes of certain foods in the human gastrointestinal tract. Still, they can only reflect the release, absorption, and distribution of food substrates after ingestion, and the results cannot fully reflect the complex physiological activities of humans [77]. Both models have their advantages and disadvantages, which need to be selected according to the needs of the actual application. It has been found that the digestibility of plant protein is about 75~80%. In contrast, animal protein has a higher digestibility of 90~95%, which is one of the important reasons why plant protein cannot completely replace animal protein [74]. The digestibility of animal and plant proteins can vary depending on the protein source, structure, type of processing, and modification. At the same time, individual differences, such as age, health status, disease status, physiological stage, and other factors, are also important factors contributing to the differences in digestibility [78]. At present, the digestibility of animal and plant proteins is mainly improved by optimising their processing techniques, such as microwave, irradiation, fermentation, and extrusion, as well as emerging processing technologies such as ultrasonic, pulsed light, high pressure, and microfiltration [78].

In addition, the dispersion, stability, bioavailability, and bioactivity of animal and plant proteins can be significantly improved through effective food encapsulation methods [79], which is also a popular technical means at present. When animal and plant protein raw materials are made into products, the dosage form of the product needs to be fully considered, which is related to whether the product can give full play to its health effects. Generally, appropriate carriers will be selected to encapsulate and deliver protein and peptide raw materials to avoid their excessive loss under unfavourable conditions, so that they can be released and further absorbed in the small intestine to exert their due health effects. In some studies, liposomes, nanoemulsions, and hydrogels are mostly used as delivery systems for protein raw materials [80]. It has been found that the soybean polypeptide-chitosan nanoparticle system based on tamarind polysaccharides and carboxymethyl cellulose can improve the release behaviour of soybean peptides in the oral cavity, has a good swelling ability, is easily eroded by saliva, has good bioaccessibility to the oral administration route, avoids intestinal and liver metabolism, and can control the release and absorption of peptides in the oral cavity [81]. In addition, the preparation of carboxymethylcellulose/polyvinyl alcohol hydrogels loaded with soybean peptides by the freeze–thaw method can overcome the barrier in the simulated gastric juice and further release the soybean peptides in the simulated intestinal fluid, effectively improving their absorption [82]. Although casein phosphopeptides (CPPs) can improve calcium absorption by inhibiting the formation of insoluble calcium deposits, peptides are easily degraded by enzymes in the stomach before entering the intestine [83], so the study of chitosan oligosaccharide nucleochitosis particles combining calcium combined with CPP for calcium delivery has shown that CPP-Ca particles can achieve a controlled calcium release and sustained calcium absorption, significantly enhancing the bioavailability of Ca [84]. The use of phytic acid, a strong crosslinker, to encapsulate the protein in chitosan microcapsules can improve its encapsulation efficiency, digestive stability, control gastrointestinal release, and oral bioavailability. The results of animal models of skin photoaging have shown that the anti-skin-photoaging and antioxidant effects of the packaging system have been significantly improved [85]. The results from the studies indicate that a suitable delivery system can improve the bioavailability of protein raw materials, improve their undesirable flavour, and maximise their health benefits. Therefore, in future research, it is worth further strengthening the research on the encapsulation technology of animal and plant proteins and peptide raw materials, to improve the bioavailability of protein raw materials and give more guarantees for their new functional applications in functional foods.

In summary, the quality of plant and animal proteins needs to be assessed in conjunction with effective ex vivo digestion models, and further research on encapsulation techniques would be beneficial for maximising the health benefits of plant and animal protein raw materials when processed into functional foods.

### 3.2. Optimising Research and Development of Animal Models

The exploration of new functions of animal and plant proteins is unknown and complex. In the process of promoting the application of animal and plant proteins and peptide raw materials in new functions, the construction of in vivo or in vitro experimental models should be strengthened, to better evaluate the possibility and mechanism of application of animal and plant proteins in new functions. At the same time, it is also more conducive to discovering the causes of certain diseases or sub-health. Preclinical in vitro and in vitro experiments provide certain safety and functional predictions for clinical research, which can greatly reduce the research cost of clinical research and can be used to derive the appropriate dosage. In addition, the evaluation results of some clinical studies are too subjective, but the evaluation can be made objectively and truthfully through preclinical in vitro and in vivo experimental studies. Although these benefits are obvious, it requires a large number of conditions to be screened, including multiple parameters such as the type of model organism, the method of modelling, the cycle, and the detection index, and it takes a lot of time and money for researchers, so the promotion of new functions is tortuous, which requires the joint efforts of multiple disciplines and scholars.

### 3.3. Increased Research into the Formulation of Animal and Plant Proteins and Peptides

There have been a lot of reports on the effects of animal and plant proteins and peptide raw materials on their own, but there are also a few studies that have found that the health benefits of combined administration are more obvious than those of a single raw material. In particular, the “double protein” combination of animal protein and plant protein raw materials has a significant synergistic effect. Whey protein and soy protein have different digestibility rates, with whey protein having a better hydrolysis rate and rapid release into plasma [86], while soy protein has a relatively slow digestibility [87], and the compatibility of the two helps to continuously supply the body’s nutritional needs and immune support. This classic “double protein” formula of animal protein + vegetable protein helps to provide rich nutrition for subjects, especially a series of active peptides that are further hydrolysed and released by a variety of proteins in the body and are more conducive to the body’s absorption and access to immune regulation [88,89]. Therefore, it is urgent that we increase the formulation research of animal and plant proteins and peptides and explore more scientific formulae to meet the health needs of the population, which is more conducive to meeting the growing needs of the market.

### 3.4. Keeping Abreast of the Latest Research Progress and Policies Related to Animal and Plant Proteins and Peptides at Home and Abroad

Product research and development are inseparable from market research and policy learning; for researchers, understanding industry policies and dynamics is the basic quality, and the release of policies for the development of the industry is huge. For example, in 2018, China issued a document to abolish the Technical Specifications for Inspection and Evaluation of Health Food (2003 Edition), which led to the reduction of the number of health food functions that can be declared in the health food industry from 27 (now 24) to 9 in the next five years, and the development of the entire industry has been greatly impacted. It was not until August 2023 that China’s State Administration for Market Regulation released the new “Methods for Functional Inspection and Evaluation of Health Foods” (2023 Edition), and the implementation of this policy has greatly activated the development of the health food market, and related scientific research results have continued to appear in people’s field of vision. At the same time, China’s State Administration for Market Regulation also issued the announcement of the “Implementation Rules for the Technical Evaluation of New Functions and Products of Health Foods (Trial)”, which provides application conditions and listing protection for the research and development of new functions of health foods, which greatly promotes the enthusiasm for the research and development of new functional raw materials of health foods, and effectively promotes the development of health food and functional food raw materials. In addition, Superbrewed Food’s microbial protein has also received Generally Recognised as Safe (GRAS) certification from the U.S. Food and Drug Administration (FDA), which is a novel, non-genetically modified organism (GMO) food ingredient that is marketed in the form of “postbiotic cultured protein” because it meets the definition of “postbiotic” proposed in the International Society for Probiotics and Prebiotics Science (ISAPP) consensus document, that is, “a formulation of inanimate microorganisms and/or their components that can provide health benefits to the host”. Not only that, but after several studies and evaluations, Superbrewed Food’s microbial protein was found to be not safe in terms of “humans, animals or the environment”, so it was added to the EUROPEAN FOODSAFETY AUTHORITY (EFSA) Safety Qualification (QPS) list in January 2024. Superbrewed Food’s microbial proteins have the health properties of postbiotics and can provide nutrients beyond protein.

In summary, researchers should pay close attention to relevant research policies at home and abroad, which are beneficial to the research and development of new functional formulations, and provide more ideas for scientific research and innovation with the help of an international perspective.

## 4. Summary and Outlook

Animal and plant proteins have shown great potential for new functions such as gut health, skin health, emotional health, oral health, and adjuvant cancer therapy, as a result of their rich composition of amino acids and active peptides. However, protein antinutrients, allergens, and unwanted metabolite raw materials have different nutrients and pollutants due to their different sources, so these unfavourable factors should be fully considered and dealt with before application, which requires a large number of basic research data to support this work, among which in vitro and in vivo digestion research, encapsulation technology research, flavour analysis and research, new functional mechanism of action research, formula research, regulatory research, and other basic research work are important means to promote the vigorous development of animal and plant proteins and peptides. At the same time, the dosage range of animal and plant proteins to exert health effects needs to be further studied, because too little protein intake will cause the body’s immunity to be impaired, and excessive protein intake will increase the burden on the kidneys, resulting in the excessive intake of sulfur-containing amino acids, which can easily accelerate the loss of calcium in bones and induce osteoporosis. Therefore, the safety and efficacy of animal and plant proteins, and the range of consumption dosages should be fully discussed. In addition, the study of the sensitisation and desensitisation mechanism of plant proteins and flavour analysis are also worthy of further discussion in the future. In short, the application of animal and plant proteins and peptides in new functions has considerable prospects, and the in-depth basic research and innovation in processing technology are necessary means to promote their development. According to the recent market data released by the Boston Consulting Group, the market size of alternative proteins is expected to reach $290 billion by 2035, of which plant-based products will account for 69% of the market, followed by microbial fermentation proteins and cell-culture proteins, which will account for 22% and 9% respectively. This suggests that researchers should consider the emerging research trend of alternative proteins in addition to the advantages of animal and plant proteins.

## Data Availability

Not applicable.
